# The MG-RAST API explorer: an on-ramp for RESTful query composition

**DOI:** 10.1186/s12859-019-2993-0

**Published:** 2019-11-08

**Authors:** Tobias Paczian, William L. Trimble, Wolfgang Gerlach, Travis Harrison, Andreas Wilke, Folker Meyer

**Affiliations:** 1 Argonne National Laboratory, Lemont, IL USA; 2 University of Chicago, Chicago, IL USA

## Abstract

**Background:**

The MG-RAST API provides search capabilities and delivers organism and function data as well as raw or annotated sequence data via the web interface and its RESTful API. For casual users, however, RESTful APIs are hard to learn and work with.

**Results:**

We created the graphical MG-RAST API explorer to help researchers more easily build and export API queries; understand the data abstractions and indices available in MG-RAST; and use the results presented in-browser for exploration, development, and debugging.

**Conclusions:**

The API explorer lowers the barrier to entry for occasional or first-time MG-RAST API users.

## Background

Environmental DNA sequence analysis (i.e., metagenomics) is gaining popularity and is frequently used by researchers who are not specialists in genomics or bioinformatics [1]. With expanding reference databases and increasing volumes of raw data, the computational component of environmental sequence analysis is substantial [2]. In metagenomic investigations, the sequence data itself is frequently in the hundreds of gigabytes range, and the datasets often reach the terabyte scale. Searching even a few metagenomes—for example, for all sequences exhibiting similarity with a set of organisms—requires either costly recomputation or storage of index data structures. Researchers handling dozens or hundreds of complex datasets frequently find themselves overwhelmed by the computational requirements of the task at hand.

Although significant advances have been made (see, e.g., [3] or [4]) that help save on computational cost, a considerable amount of compute, storage, and I/O bandwidth is required to analyze metagenomic data. Projects that must analyze many datasets across thousands of directories and terabytes of data often reach the limit of on-premise or temporary rented public cloud resources. Consequently, remote computation, data management, and indexing of large-scale metagenomic data are a growing community need and will be key features of any future bioinformatics landscape.

Hosted services such as MG-RAST [5], the EBI Metagenomics Portal [5], and the U.S. Department of Energy’s JGI IMG/M [6] provide web interfaces to access the data, computational results, and search results. However, these interfaces often are limited to predefined queries, even if the underlying data and indexing support additional query capabilities. Exposing the internal data and indices via an application programming interface (API) helps overcome this limitation, enabling end users potentially to delve more deeply into the data.

In addition, studying larger quantities of datasets is often done best via custom scripts or command line tools. APIs play an essential role by allowing automation. We also note that APIs render the practice of extracting data from web pages (“screen scraping”) obsolete.

In recent years RESTful APIs [7] have become the state of the art, allowing data to be managed and distributed over the internet. These APIs now provide the backbone of commerce and entertainment on the internet, and they have become a necessary tool for the handling of sequencing data and data products.

For example, MG-RAST is designed around a RESTful API [8]. This provides a search engine for datasets, delivers tables of taxa and functional annotations from sequence datasets, allows upload and download of data files, and can deliver sequences with attached annotations. Structured data is delivered in the JSON format; JSON data can be easily converted to tab-delimited tables or other formats used in bioinformatics if needed. While JSON is not particularly user friendly it has emerged as the standard and a myriad of tools exist to assist.

Unfortunately, although RESTful APIs offer more flexibility to access computation and data, they are notoriously difficult to learn [9]. Two factors contribute to this steep learning curve: the syntax and the abstractions. Syntax refers to the fact that APIs are intended to be consumed primarily by computer programs, not human beings, and require strict adherence to standards and conventions. Abstractions refer to the fact that APIs require some level of understanding of the abstractions used for data storage and indexing. However, when a query against a readily indexed database of metagenomic data can save tens of thousands of dollars of computational (and manpower) costs for reanalyzing metagenomic datasets, learning to work with RESTful APIs becomes an attractive value proposition.

### Implementation

The API explorer is implemented as light-weight JavaScript and HTML overlay on top of the MG-RAST API [8] and the MG-RAST infrastructure [10]. The API itself is supported by a complex mixture of databases and object stores.

Like all of MG-RAST, the API explorer is available as open source software on GitHub at https://github.com/MG-RAST under a BSD-style license.

## Results

To assist researchers in learning the syntax of the MG-RAST RESTful API, the MG-RAST team has developed an API explorer (https://explorer.mg-rast.org/). It has one page for search and one for all other API functionality. The explorer allows querying capabilities and displays the results of an API query in-browser. Additionally, it constructs working command line invocations that can be copied and pasted.

The MG-RAST API explorer provides a gentle introduction to the API through a number of simple example queries.

**Fig. 1:**
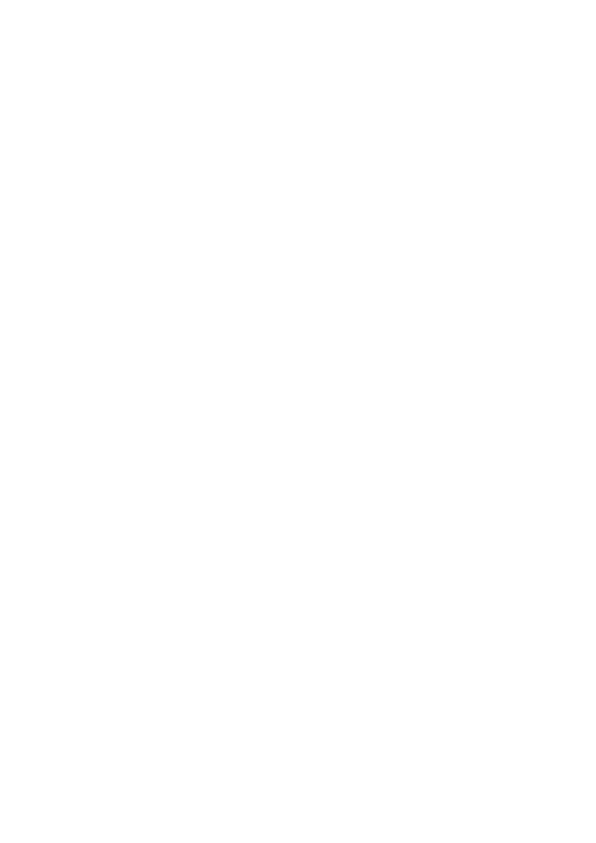
URL and the results for the retrieval of SwissProt taxonomy annotations with a cut-off 10^− 10^ for dataset mgm4447943.3

**Fig. 2:**
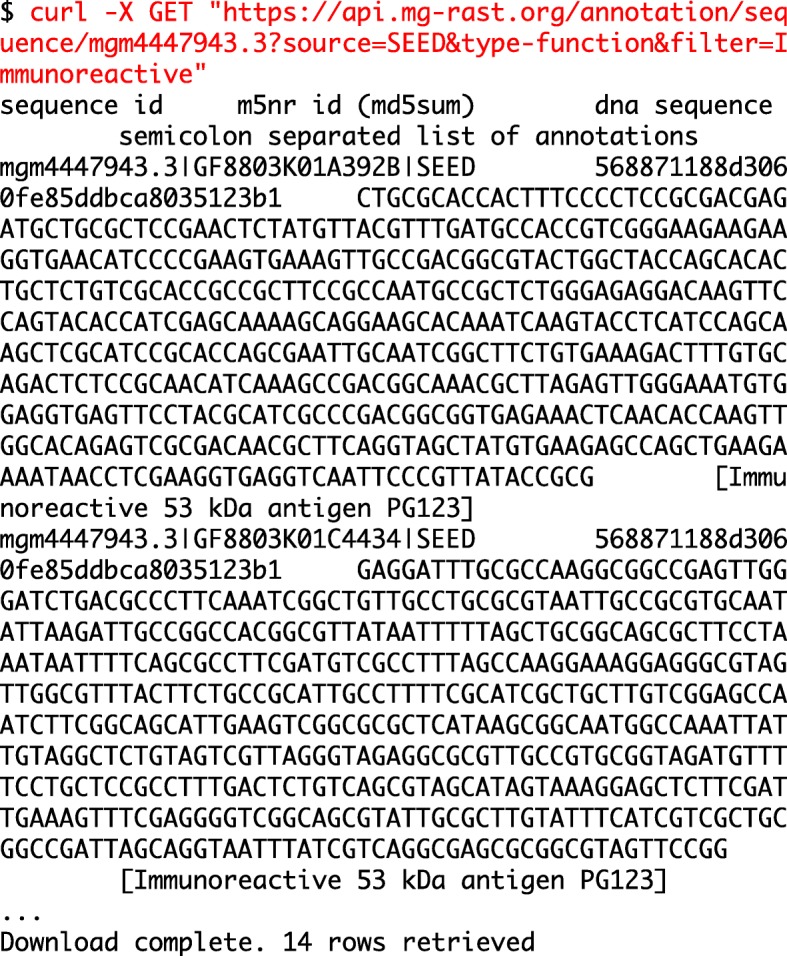
URL and the results for the retrieval of SwissProt taxonomy annotations with a cut-off 10− 10 for dataset mgm4447943.3

**Fig. 3:**
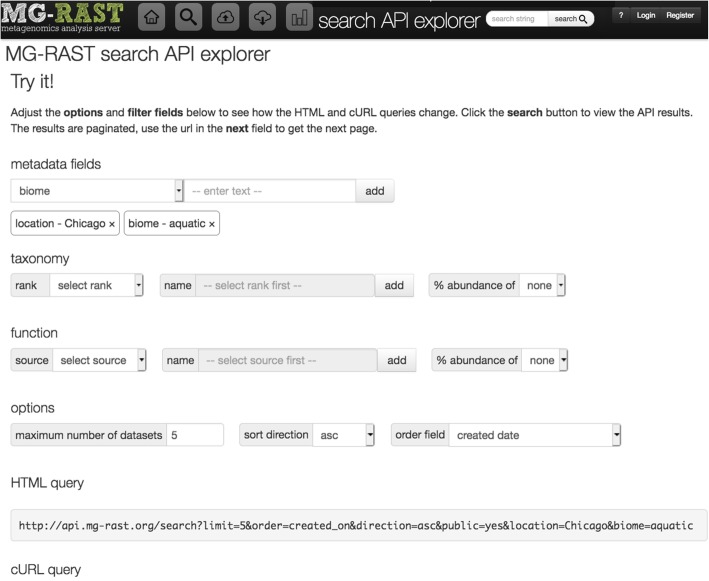
Using the API explorer to construct a query for aquatic datasets from Chicago. This query was built by selecting “location” from a drop-down menu, entering the value “Chicago,” and selecting “biome” from the same menu and entering the term “aquatic.” All the valid API options are presented as editable fields; the API’s response to the query is shown in the gray box at the bottom of the page labeled “result from API.”

**Fig. 4:**
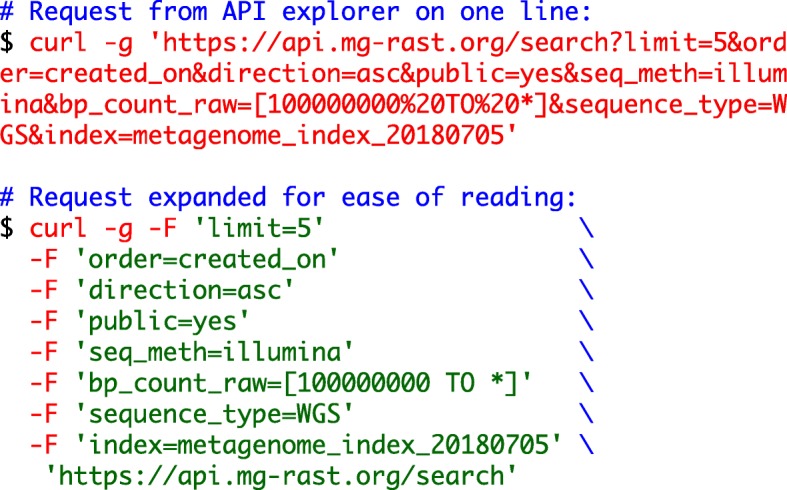
URL and command line syntax for retrieving a list of Illumina shotgun metagenomes larger than 1 gigabase pair. This query returns a list of matching datasets in JSON format

**Fig. 5:**
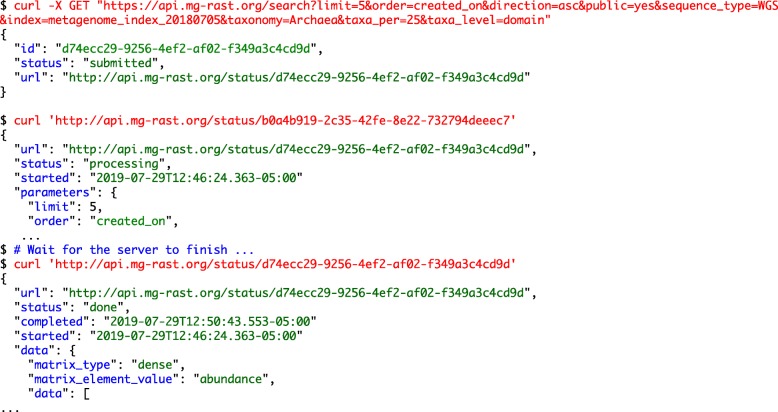
Searching for datasets with more than 25% Archaea. This complex search request will not return results immediately. In order to avoid client-side timeouts, the queries are turned into asynchronous search requests. The output of the curl command will indicate this and provide a URL for status checking on the complex queries and eventual download. We note that the URL contains a UUID to act as a temporary identifier

- **Example 1: Annotated sequence retrieval.** Downloading annotated sequences requires specifying a dataset, a database (one of the databases included in the union M5nr database [8]), and cut-off thresholds for the similarity table. Indexes for both taxonomy (Fig. 1) and functions (Fig. 2) allow retrieval of annotated sequences with organism name or function labels attached.
- **Example 2. Query composition.** The next example involves a simple query requesting a list of aquatic datasets collected in Chicago. Figure 3 shows the URL and command line representation generated by the API explorer.
- **Example 3: Dataset properties.** Searches for datasets can also include technical properties such as dataset size or type. This example retrieves all shotgun metagenomic datasets using Illumina technology larger than 1 gigabase pair. The resulting URL and command line are given in Fig. 4.
  In addition to storing metadata about the datasets, MG-RAST stores the results of the sequence analysis, indexed to allow querying by organism name (such as *Corynebacterium glutamicum*), taxonomic names from the NCBI taxonomy, or protein function labels from the included protein function databases. See [9] for details of the computational pipeline.
- **Example 4: Dataset content.** The MG-RAST RESTful API explorer can also be used to find datasets based on dataset content rather than metadata. In this example we retrieve a list of datasets with a substantial fraction of sequences annotated as Archaea and later aggregate the abundance information at the family level. The query in Fig. 5 returns datasets with more than 25% of the sequences showing protein similarities to Archaea. We note that the API returns structured data in JSON format and abundance tables in BIOM format [10]. While JSON is not particularly readable, numerous tools are available for converting JSON into other formats, for example, CSV for use in spreadsheets.

**Fig. 6:**
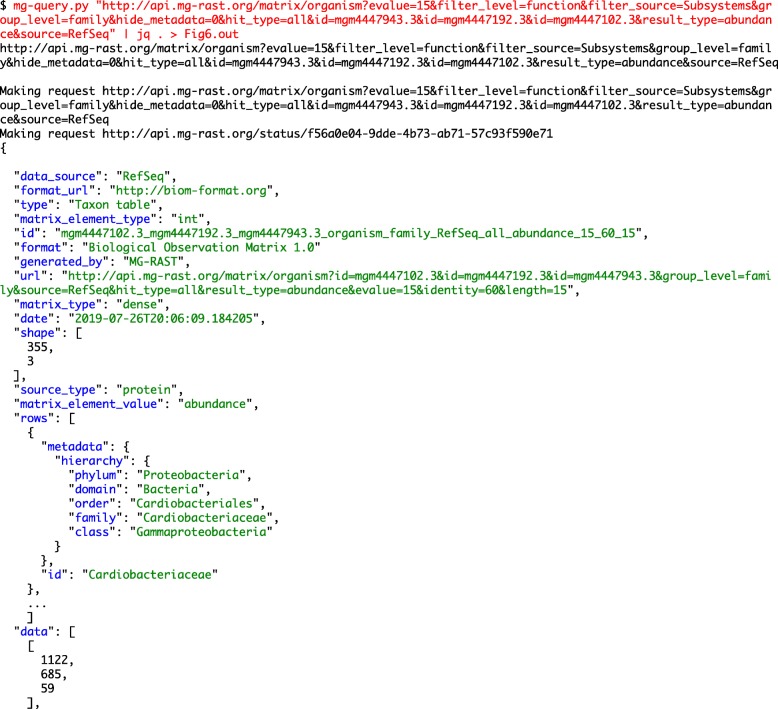
The URL constructed via the API explorer generates an abundance count of SEED subsystem terms for 3 datasets, summarizing abundance at the family level using RefSeq generated taxonomic annotations. The output is again in JSON format; we show the top of the return file using jq to color code it for readability

The matrix function of the API explorer allows for merging information on taxonomy and function (see Fig. 6). Moreover, the API is not limited to abundance and taxonomy tables; sequences labeled with unstructured, free-text functional annotations from included databases can also be listed or extracted (see Fig. 2).

- **Example 5: Sequence retrieval.** For our next example, the API retrieves sequences, decorated with annotations, that match specified organisms or functions: the sequences returned by this URL all have similarity to “Immunoreactive proteins” in the SEED database.
- **Example 6: Metaanalysis**-extracting GPS coordinates. With thousands of data sets available in MG-RAST, metaanalyses are becoming more popular. The API supports data extraction and analysis that can be used to explore the coverage of the planet with metagenomic samples.

**Fig. 7:**
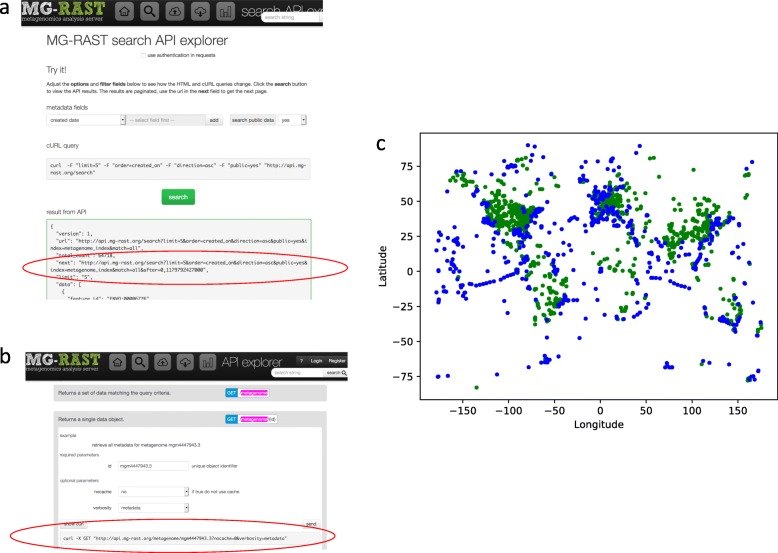
Illustration of using the API Explorer to build queries for metaanalysis. **a**) Search API guides the construction of queries for lists of metagenomes **b**) The API Explorer guides building the sub-queries for rich data about each public dataset. A subset of this data bundle is the latitude and the longitude, which can be **c**) visualized

## Conclusion

All the working examples shown here used public data and did not require authentication. However, private datasets can be securely accessed by adding “&auth = MGRKEY” to URLs used in any browser or – H “Authorization: mgrast MGRKEY” to the command line for curl, where MGRKEY is replaced by the text of the MG-RAST authentication key, a password-like string that is available from the user’s upload page.

We also provide a set of client-side Python scripts to assist with standard use cases, allowing, for instance, data upload and download without using the browser and automatically handling authentication and waiting for long-running queries; see the website. https://github.com/MG-RAST/MG-RAST-Tools.

The MG-RAST team hopes that the drop-down menus and in-browser troubleshooting environment provided by the MG-RAST RESTful API explorer will help researchers make better use of the metagenomic data and computation already completed and curated at MG-RAST (Fig. 7). The MG-RAST system is open source and is available on github (https://github.com/MG-RAST).

## Availability and requirements

The API explorer is available as part of MG-RAST.

**Project name:** MG-RAST

**Project home page:** https://github.com/MG-RAST

**Operating system(s):** Linux

**Programming language:** Perl, Python, Go-Lang, HTML5

**Other requirements:** ElasticSearch, Cassandra, SOLR, MongoDB, SHOCK, AWE

**License:** BSD type license

**Any restrictions to use by non-academics:** none.

## Data Availability

The source code is available on github under a BSD license. All data used in the examples is publicly available on MG-RAST.

## Abbreviations

API: Application programmer’s interface
BIOM: Biological Observation Matrix
I/O: Input and output and movement of computer data
JSON: Javascript object notation
NCBI: National Center for Biotechnology Information
URL: Uniform resource locator
